# Lomustine Analogous Drug Structures for Intervention of Brain and Spinal Cord Tumors: The Benefit of In Silico Substructure Search and Analysis

**DOI:** 10.1155/2013/360624

**Published:** 2013-04-16

**Authors:** Ronald Bartzatt

**Affiliations:** University of Nebraska, College of Arts & Sciences, Durham Science Center, 6001 Dodge Street, Omaha, NE 68182, USA

## Abstract

Lomustine is a nitrosourea anticancer agent shown to be effective for treatment of childhood medulloblastoma. In silico substructure searches produced 17 novel nitrosourea agents analogous to lumustine and retaining activity for DNA alkylation and cytotoxic activity. The mean values for Log *P*, polar surface area, formula weight, number of oxygens & nitrogens, and rotatable bonds were 2.524, 62.89 Anstroms^2^, 232.8, 5, and 2, respectively. All 17 agents have formula weight less than 450 and Log *P* less than 5, two criteria preferred for blood-brain barrier penetration. These agents have a polar surface area less than 90 Angstroms^2^. Each show zero violations of the Rule of five indicating favorable drug likeness and oral drug activity. Hierarchical cluster analysis indicated that 16 of the novel agents were highly similar to lomustine, save for agent 12 which bears a hydroxylated branched carbon substituent. A total of 17 novel anticancer agents were elucidated having molecular properties very effective for penetrating through the BBB and into the central nervous system. This study shows the effectiveness of in silico search and recognition of anticancer agents that are suitable for the clinical treatment of brain tumors.

## 1. Introduction

 Tumors of the brain and spinal cord are considered the third most common type of childhood cancers with only leukemia and lymphoma having greater occurrence. Tumors that occur in the central nervous system (CNS) can be either primary (tumors that originate in the CNS) or metastatic (tumors formed from cancer cells having origins in other parts of the body). The various types of childhood spinal cord and brain tumors include the following: astrocytomas, atypical teratoid tumor, brain stem glioma, CNS embryonal tumor, CNS germ cell tumor, craniopharyngioma, ependymoma, medulloblastoma, spinal cord tumors, and supratentorial primitive neuroectodermal tumors [1]. 

 The metastases-type tumors are the most common type of cancer of the CNS and appear to be increasing in incidence [2]. The pathophysiology of the brain in which metastases occur is very important for it is the location of the tumor that can lead clinicians to apply more effective therapies to target tumor growth [2].

Clinical studies conducted in Korea have shown that females are more inclined to CNS tumors (at a ratio of 1.43 : 1) and with the most common tumor type to be meningioma (31.2%), followed by glioblastoma (30.7%), and finally malignant primary tumors (19.3%) [3]. For childhood aged cases (these being less than 19 years of age) the most common types are germ cell tumors and embryonal-medulloblastoma [3]. Therapies for younger children having medulloblastoma include the use of multiagent chemotherapeutic approaches [4]. For children older than 3 years having nondisseminated disease and for partially resected high-risk disease, the standard therapy includes both treatment with radiotherapy and adjuvant chemotherapy [5]. Most therapeutic approaches have focused on either delaying or eliminating radiotherapy by the use of increasingly aggressive chemotherapeutic approaches [6].

 The design of novel drugs to treat CNS-located tumors should be focused on agents having useful antitumor activity in addition to the capability of crossing the blood-brain barrier (BBB) [7]. Children of all ages are susceptible to the adverse effects of radiation on brain development. Results suggest that chemotherapy can be used to delay and sometimes obviate the need for radiation therapy in 20% to 40% of children younger than 3 years with nondisseminated medulloblastoma [8–10]. The appearance of brain metastases occurs in up to 40% of cancer patients with this incidence increasing in frequency [11]. 

 Children diagnosed with neuroectodermal tumor of the cerebella, referred to as medulloblastoma, have a poor prognosis [12]. However, when adjuvant chemotherapy including lomustine, cisplatinum, and vincristine is applied following radiotherapy, the rate of relapse is reduced from 100% to 11.1% [12]. Other clinical findings show that when taken individually, lomustine, vincristine, cisplatin, and cyclophosphamide confer the most beneficial survival effects over craniospinal radiotherapy alone [13]. Similarly, lomustine (and procarbazine) was shown to have marked inhibition of neuroectodermal tumor, ependymomas, medulloblastoma, and astrocytic gliomas in another study [14]. These studies encourage and support the consideration and design of antineoplastic agents that are structural analogs of lomustine, but also for comparison to established guidelines of molecular properties determined to be clearly efficacious for penetrating through the BBB and entering the central nervous system. 

 Computational in silico methods are widely applied to pharmacology hypothesis development and testing [15]. In silico methods are frequently applied in the discovery and optimization of novel molecules that have exclusive affinity for a particular target. In addition, in silico methods are used for elucidation of absorption, distribution, metabolism, excretion, and toxicity properties of proposed medicaments as well as the determination of physicochemical properties [15]. 

 Therefore, clinical studies clearly reveal the need for novel antitumor agents that have effective antineoplastic activity but with molecular properties enabling the penetration of the CNS. Although difficulties of CNS penetration are substantial due to the BBB, design of molecular structures that effectuate CNS infiltration is vital for treatment of brain tumors.

## 2. Materials and Methods

### 2.1. Molecular Modeling

 Visualization and modeling of 2-dimensional and 3-dimensional structures were accomplished by ACD Chem Sketch modeling v.10.00 (Advanced Chemistry Development, 110 Yonge Street, Toronto, ON, Canada M5C1T4). Various properties such as polar surface area, violations of Rule of 5, molecular volume, number of oxygens, nitrogens, amines, and hydroxyls were determined using Molinspiration (Molinspiration Cheminformatics, Nova ulica 61, SK-900 26 Slovensky Grob, Slovak Republic). In silico structure search for substituent replacement was accomplished using chemical substructure and similarity search with Molinspiration Molecular Database-Substructure and Similarity Search (http://www.molinspiration.com/services/search.html).

### 2.2. Pattern Recognition Techniques

To identify underlying associations and patterns within the multivariate data set required the use of various pattern recognition techniques. Included in the analysis is hierarchical cluster analysis accomplished by KyPlot v. 2.0 Beta 15 (copyright Koichi Yoshioka 1997–2001). Analysis of similarity (ANOSIM) is accomplished by PAST v. 2.04 (copyright Oyvind Hammer, D.A.T. Harper 1999–2008). Path analysis was accomplished utilizing OpenStat numerical analysis (copyright W. Miller November 20, 2010).

### 2.3. Numerical Analysis

Summary statistical analysis of molecular properties of numerical data including correlation analysis for Pearson *r* was performed by Microsoft EXCEL (EXCEL 2003, copyright 1985–2003). Multiple regression analysis of molecular properties was accomplished by Graph Pad Instat v. 3.00 for Windows 95 (Graph Pad Software, San Diego, CA, USA). Grubbs' test, also called the ESD method (extreme studentized deviate), to determine whether the most extreme value in the list entered is a significant outlier from the rest (whether that one value is an outlier) was determined by GraphPad online Software (GraphPad Software Inc. 2236 Avenida de la Playa, La Jolla, CA 92037, USA; http://www.graphpad.com/quickcalcs/grubbs2/). 

## 3. Results and Discussion

 Numerous studies have demonstrated that rigorous criteria of molecular properties strongly correlate with and define medicaments that penetrate through the BBB and into the CNS. Outcomes of such studies report criteria for effective CNS penetration by medicaments having polar surface area (PSA) of 90 Angstroms^2^ or less and formula weight cutoff of 450 [16]. Additional studies encompassed other molecular properties to expand the criteria. Contemporary studies indicate that penetration into the CNS is most likely if the following criteria exist [17]: (1) the formula weight is less than or equal to 400; (2) Log *P* is less than or equal to 5; (3) hydrogen bond donors (–NH_*n*_ and –OH) less than or equal to 3; and (4) hydrogen bond acceptors (oxygen and nitrogen) less than or equal to 7. All 17 of the novel drugs proposed here meet or exceed these necessary criteria for penetration through the BBB.

**Figure 1:**
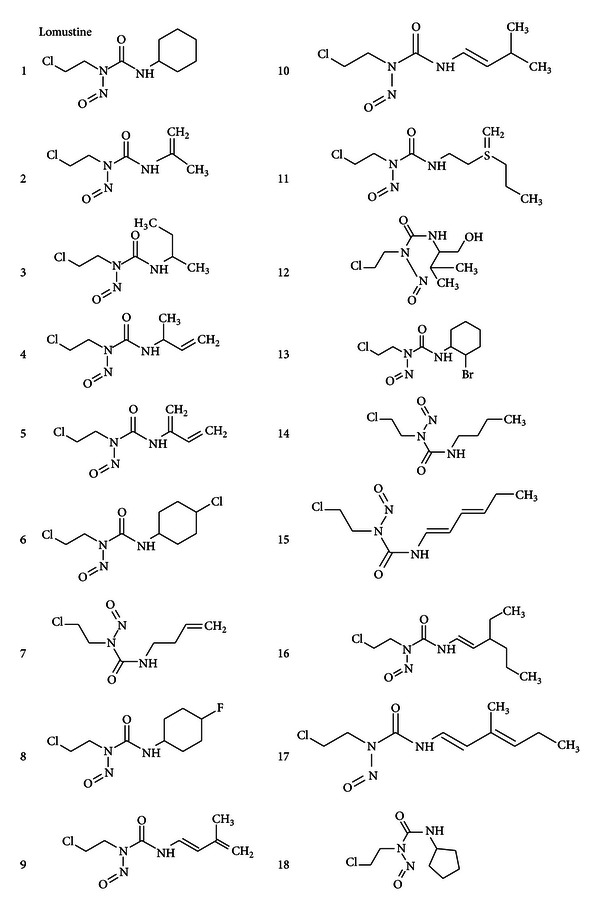
Seventeen novel nitrosourea anticancer structures (2 to 18) obtained from substructure and similarity search analysis applying lomustine as parent structure are presented for comparison. The structures are substantially diverse by substituent following the nitroso (R–NO) and urea group OC(NH_*n*_)_2_. Other notable substituent aspects include aliphatic carbon chains, double-bond carbon chains, hydroxyl (–OH) groups, halogens (Cl and Br), and ring structures.

 Presented here are 17 novel drug designs elucidated by in silico search by way of substituent similarity and substitution utilizing lomustine as the parent structure. Accomplished through Molinspiration data base library,these 17 agents were recognized from a total of more than 200 generated structures, thus having a success outcome of less than 10%. The 17 novel compounds identified (agents 2 to 18) are presented in Figure 1 for a comparison with the parent compound which is lomustine (agent 1). 

 Notable structural characteristics must include the alkylating and cytotoxic nitrosourea moiety (O=C(NHR–)N(N=O)CH_2_CH_2_Cl), with (–R) defined as the substituent providing the variation of molecular properties (e.g., Log *P*, PSA, formula weight, etc.) but constrained to enhance penetration into the CNS. 

The (–R) substituent for lomustine is the nonaromatic ring C_6_H_12_. A wide variety of structural substituents include: halogens (agents 6 and 13), aliphatic carbon chains (14,3), alkene carbon chains (17, 16, 15, 7, 5, 10, 4), hydroxyl groups –OH (14), carbon rings (8, 13, and 18), and a sulfanylidene group (11). The marked variation in the (–R) substituent is shown to enable a beneficial variation in molecular properties of vital significance to effectuate drug penetration into the CNS.

 Pharmacological properties of agents 1 to 18 are compiled in Table 1. Careful statistical analysis of numerical values is shown in Table 2 for consideration of library search efficiency. Notably the mean Log *P* is 2.524 (standard deviation (SD) = 0.5337) with range of 1.537 to 3.924: formula weight (FW) mean of 232.8 and range of 191.6 to 312.6. The range for number of hydrogen bond acceptors oxygen and nitrogen atoms (O & N) is tight from 5 to 6, mean of 5. Likewise the range for hydrogen bond donors hydroxyl (–OH) and amines (–OH & –NH_*n*_) is only 1 to 2 with mean of 1. These four properties are selected outright to emphasize that the search outcome adheres very well with the CNS penetrating criteria stated previously to wit [17]: (1) FW ≤ 400; (2) Log *P* ≤ 5; (3) –OH &–NH_*n*_ ≤ 3; (4) O & N ≤ 7. Similarly, another criterion of PSA ≤ 90 Angstroms^2^ and FW ≤ 450 [16] is well satisfied for BBB penetration, from numerical values in Table 1. Clearly the in silico library search for similarity and substituent substitution can be highly accurate; even the criteria are highly precise in demand. Formula weight was highly correlated (Pearson *r* > 0.8700) to number of atoms, and molecular volume was highly correlated to number of atoms and formula weight (*r* > 0.8700). The number of atoms, molecular volume, and formula weight is moderately correlated to Log *P* (Pearson *r* > 0.5000). There are no outliers (two-sided *P* = 0.05) among numerical values of Log *P*, formula weight, and molecular volume.

 Multivariate statistics is a form of statistics encompassing the simultaneous observation and analysis of more than one outcome variable (see Table 1) [18].

**Figure 2:**
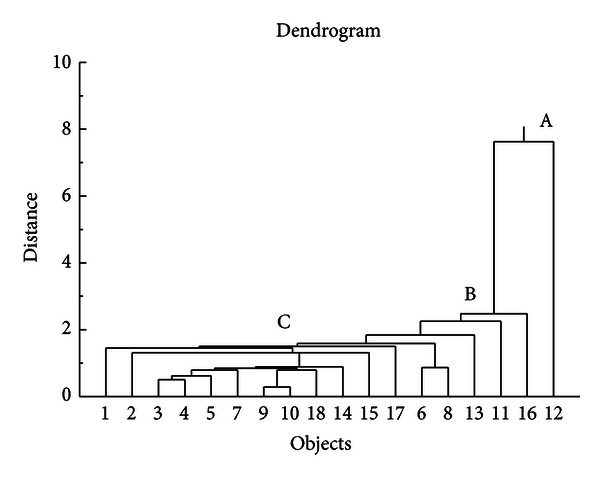
Result of hierarchical cluster analysis (Euclidean distance, single linkage) presented as vertical dendrogram and divisive analysis is presented. The initial supercluster is separated into a unique distinction of agent 12 at node A, followed with others 16, 11, 13, 6, and 8 at node B, finally including lomustine (1) at node C.

The purpose of cluster analysis is to discover a system of organizing objects into groups (or clusters) where members within the groups share properties in common suggested by the data itself (i.e., not known a priori), and objects in different clusters tend to be dissimilar [18]. Hierarchical cluster analysis of the multivariate Table 1 produced the vertical divisive dendrogram presented in Figure 2 for visualization of the relative resemblance to the parent compound lomustine (agent 1). Conditions are standard Euclidean distance (the geometric distance in the multidimensional space) and single linkage (the distance between two clusters is determined by the distance of the two closest objects) [18]. The analysis clearly distinguishes agent 12, at node A (containing a hydroxyl group –OH), from all the remaining agents. Essentially all remaining agents fall under node B, but includes some finer elucidation of agents 16, 11, 13, 6, and 8 from the remainder under node C. Other than agent 12, the finer closest similarity of lomustine is to agents 2, 3, 4, 5, 7, 9, 10, 18, 14, 15, and 17. 

 Additional advantage of these nitrosourea agents is properties that affirm effective oral administration. Oral activity is a desirable druglikeness character that is identified through zero violations of the Rule of 5 [19]. The rule states that, in general, an orally active drug has no more than one violation of the following criteria: (1) not more than 5 hydrogen bond donors (nitrogen or oxygen atoms with one or more hydrogen atoms); (2) not more than 10 hydrogen bond acceptors (nitrogen or oxygen atoms); (3) a molecular mass less than 500 daltons; and (4) an octanol-water partition coefficient Log *P* not greater than 5. 

 Previous studies have shown that PSA and Log *P* can be used to estimate the quantitative transfer of a neurological drug from the blood into the CNS.

The ratio of drug concentration in the brain to concentration in the blood can be represented as *C*_brain_/*C*_blood_ or BB. The expression utilizing PSA and Log *P* to accurately determine Log BB [20] is shown in

(1) $$\begin{array}{c} \text{Log}\;\text{BB}=-0.0148\left(\text{PSA}\right)+0.152\left(\text{Log}\;P\right)+0.139. \end{array}$$

For agents 1 to 18, the values of Log BB and BB are shown in Table 3. 

The average value of BB (or *C*_brain_/*C*_blood_) is 0.405 (standard  deviation = ± 0.102) with minimum of 0.144 and maximum of 0.662. The average value of Log BB is −0.108 compared to Log BB of lomustine at −0.325. These results indicate that a significant amount of each agent will cross into the CNS. No outlier was found among numerical values of BB (two-sided *P* = 0.05). 

 The purpose of multiple regression is prediction of a dependent variable based on multiple independent variables [18] or molecular properties for drug prediction. Multiple regression analysis of descriptors in Table 1 to forecast formula weight (FW) of similar compounds based on Log *P*, PSA, number of rotatable bonds (nRot), and number of atoms (Atoms) the following equation (2) accounts for 79.34% of the variance in formula weight (*R* ^2^ = 0.7934):

(2) FW=−24.859+0.9578(Log P)−0.04598(PSA)+19.236(Atoms)−3.947(nRot).

ANOSIM (Analysis of Similarity) is a nonparametric (randomization-based) method of multivariate analysis that is widely used. It is used mainly to compare the variation of object abundances and composition among sampling units [21].

ANOSIM result for Table 1 multivariate array is *R* = 1.000, which suggests a diverse distribution of numerical values within these properties and a characteristic of the in silico search result. ANOSIM results reaffirm the diverse nature of the structure-based substituent substitution with differences of atoms and their effect on molecular properties.

 Path analysis is a straightforward extension of multiple regression analysis also for prediction. The aim is to provide estimates of the magnitude and significance of hypothesized causal connections among sets of variables [21]. Path coefficients are standardized weights which can be used in examining the possible causal linkage between variables. A path coefficient shows the direct effect of an independent variable on a dependent variable in the path model [21]. 

Path coefficients for causal relationship of various properties to formula weight are shown in Table 4. The greater the positive value of the path coefficient, the stronger the causal relationship. Clearly the number of atoms (coefficient = 0.449) and number of rotatable bonds (coefficient = 0.499) have a level of moderate to strong causality for formula weight, Unlike Log *P*, PSA, molecular volume, number of –OH and –NH_*n*_, and number of O and N atoms that have coefficient values less than 0.100. 

 In silico pharmaceutical modeling is ongoing and demonstrates plentiful array of possibilities in expediting the discovery of new targets and discerning lead compounds that have predicted biological activity for these novel targets [15].

In silico methods include databases, quantitative structure-activity relationships, similarity searching, pharmacophores, homology models, molecular modeling, machine learning, data mining, network analysis tools, and computation of based data analysis tools [15]. The cultivation of brain tumor biopsies has been achieved [22], and the testing of these nitrosourea agents and other similar anticancer drugs could be evaluated for effectiveness by in vitro methods.

## 4. Conclusions

 In silico substructure search produced 17 novel nitrosourea agents that are analogous to lomustine and retain the DNA alkylating component required for cytotoxic activity. The 17 new designs were derived from a data library search netting over 200 hits but only the structures presented here have molecular properties suitable for penetration through the BBB and into the CNS. This is a success rate of less than 10% of all search excerpts. All compounds have a polar surface area of less than 90 Angstroms^2^ and Log *P* values of less than 5. 

 Prediction of drug passage into the CNS can be quantitatively estimated using relationships of PSA and Log *P* from studies of current neurological drugs. For these 18 nitrosoureas, the mean values of Log BB and BB are −4.08 and 0.405, respectively. No outlier was found among values of BB (two-sided *P* = 0.05). 

The range in BB values extended from 0.144 to 0.662, showing that estimated levels of CNS penetration are significant for all structures. 

 The use of in silico methods is a potent and promising addition to other approaches to drug candidate elucidation. The database generated 17 nitrosourea agents having zero violations of the Rule of 5 as well as molecular properties known to enable and enhance penetration of the CNS. This study shows that a contemporary algorithm for searching molecular libraries is able to identify structures suitable for further examination. The molecular properties necessary for effective neurological activity are sufficiently elucidated for identifying potential candidates that fall within the rigid criteria for clinical application.

## Figures and Tables

**Table 1:** Molecular properties of anticancer agents.

Drug	Log *P *	Polar surface area (Angstroms^2^)	Number of atoms	Molecular weight	Number of O & N	Number of –OH and –NH_*n*_	Violations of Rule of 5	Number of rotatable bonds	Volume (Angstroms^3^)
1	2.965	61.772	15	233.7	5	1	0	4	208.8
2	2.188	61.771	12	191.6	5	1	0	4	163.1
3	2.268	61.771	13	207.7	5	1	0	5	185.5
4	2.034	61.771	13	205.6	5	1	0	5	179.9
5	2.457	61.771	13	203.6	5	1	0	5	174.2
6	2.583	61.771	16	268.1	5	1	0	4	222.3
7	1.975	61.771	13	205.6	5	1	0	6	180.1
8	2.276	61.771	16	251.7	5	1	0	4	213.7
9	2.429	61.771	14	217.7	5	1	0	5	190.5
10	2.359	61.771	14	219.7	5	1	0	5	196.1
11	2.476	61.771	16	267.8	5	1	0	8	236.9
12	1.537	81.999	15	237.7	6	2	0	6	210.4
13	3.184	61.771	16	312.6	5	1	0	4	226.7
14	2.498	61.771	13	207.7	5	1	0	6	185.7
15	2.635	61.771	15	231.7	5	1	0	6	206.9
16	3.924	61.771	17	261.7	5	1	0	8	246.5
17	3.182	61.771	16	245.7	5	1	0	6	223.5
18	2.459	61.771	14	219.7	5	1	0	4	191.9

**Table 2:** Property statistics.

Property	Mean	Minimum	Maximum	Median	Standard deviation
Log *P *	2.524	1.537	3.924	2.458	0.5337
Polar surface area (A^2^)	62.89	61.77	82.0	61.77	4.768
Number of atoms	14.5	12	17	14.5	1.465
Molecular weight	232.8	191.6	312.6	225.7	30.76
Number of O & N	5	5	6	5	0.2357
Number of –OH and –NH_*n*_	1	1	2	1	0.2357
Violations of Rule of 5	0	0	0	0	0
Number of rotatable bonds	2.3	4	8	5	1.274
Volume (A^3^)	202.4	163.1	246.5	201.5	23.03

**Table 3:** Log BB and BB values.

Agent	Log BB	BB
1, lomustine	−0.325	0.473
2	−0.443	0.361
3	−0.430	0.372
4	−0.466	0.342
5	−0.402	0.396
6	−0.383	0.414
7	−0.475	0.335
8	−0.429	0.372
9	−0.406	0.393
10	−0.416	0.383
11	−0.399	0.399
12	−0.841	0.144
13	−0.291	0.512
14	−0.396	0.402
15	−0.375	0.422
16	−0.179	0.662
17	−0.292	0.511
18	−0.401	0.397

**Table 4:** Path analysis causal effect on formula weight.

Property	Path coefficient
Log *P *	−0.085
Polar surface area	−0.027
Number of atoms	0.449
Number of oxygens and nitrogens	−0.027
Number of –OH and –NH_*n*_	−0.026
Number of rotatable bonds	0.499
Molecular volume	0.005
